# Brassinazole resistant 1 (BZR1)-dependent brassinosteroid signalling pathway leads to ectopic activation of quiescent cell division and suppresses columella stem cell differentiation

**DOI:** 10.1093/jxb/erv316

**Published:** 2015-07-01

**Authors:** Hak-Soo Lee, Yoon Kim, Giang Pham, Ju Won Kim, Ji-Hye Song, Yew Lee, Yong-Sic Hwang, Stanley J. Roux, Soo-Hwan Kim

**Affiliations:** ^1^Division of Biological Science and Technology, Yonsei University, Wonju, 220–710, Republic of Korea; ^2^Department of Life Science, Hanyang University, Seoul, 133–791, Republic of Korea; ^3^Department of Bioscience and Biotechnology, Konkuk University, Seoul 143–701, Republic of Korea; ^4^Department of Molecular Biosciences, University of Texas, Austin, TX 78712, USA

**Keywords:** Auxin, brassinosteroid, BZR1, columella stem cell, PINs, quiescent centre (QC), root apical meristem, root development.

## Abstract

Previous publications have shown that BRI1 EMS suppressor 1 (BES1), a positive regulator of the brassinosteroid (BR) signalling pathway, enhances cell divisions in the quiescent centre (QC) and stimulates columella stem cell differentiation. Here, it is demonstrated that BZR1, a BES1 homologue, also promotes cell divisions in the QC, but it suppresses columella stem cell differentiation, opposite to the action of BES1. In addition, BR and its BZR1-mediated signalling pathway are shown to alter the expression/subcellular distribution of pin-formed (PINs), which may result in changes in auxin movement. BR promotes intense nuclear accumulation of BZR1 in the root tip area, and the binding of BZR1 to the promoters of several root development-regulating genes, modulating their expression in the root stem cell niche area. These BZR1-mediated signalling cascades may account for both the ectopic activation of QC cell divisions as well as the suppression of the columella stem cell differentiation. They could also inhibit auxin-dependent distal stem cell differentiation by antagonizing the auxin/WOX5-dependent pathway. In conclusion, BZR1-/BES1-mediated BR signalling pathways show differential effects on the maintenance of root apical meristem activities: they stimulate ectopic QC division while they show opposite effects on the differentiation of distal columella stem cells in a BR concentration- and BZR1-/BES1-dependent manner.

## Highlight

Brassinosteroids (BRs) lead to ectopic activation of quiescent centre division as well as modulation of the columella stem cells differentiation in *Arabidopsis* roots in a BR concentration- and BZR1-/BES1-dependent manner.

## Introduction

The apical regions of plant roots are composed of mitotically inactive quiescent centre (QC) cells and the surrounding meristemic stem cell initials (epidermal-lateral root cap initials, cortical-endodermal initials, stele initials, and columella initials), which form a ‘stem cell niche’ (SCN) (Supplementary Figure S1 available at *JXB* online; Bennett and Scheres, 2010). Each initial cell gives rise to the different root tissues, being the outermost lateral root cap, epidermis, ground tissue (cortex and endodermis), pericycle, and the innermost vascular tissues to make up a radial arrangement of root tissues. Along the longitudinal axis, the root is composed of a distal structure [lateral root cap and columella layers derived from the columella stem cell initials (CSCs)], the SCN, proximal meristem, transition zone, elongation zone, and differentiation zone (Perilli and Sabatini, 2010; Lee *et al.*, 2013). This cylinder-like concentric root structure is established by a balance between cell division in initials, stem cells, and meristematic cells of the proximal/distal meristems, and differentiation of these cells into diverse cell types in the elongation/differentiation zone. This balance is under the tight control of unknown QC-directed signals and environmental cues that regulate the spatiotemporal expression of master regulatory genes for cell fate determination (Scheres, 2007; Bitonti and Chiappetta, 2011). In general, when asymmetric division of a stem cell initial occurs, the daughter cell that has contact with the QC remains as an initial cell whereas the other daughter cell—which is separate from the QC—divides to form transit-amplifying cells at the boundary of the proximal meristem (Scheres, 2007; Perilli *et al.*, 2012).

Many hormones and protein factors have been shown to be involved in the specification and maintenance of QC and stem cell identity, and the resulting determination of radial and longitudinal patterning (reviewed in Bennett and Scheres, 2010; Bitonti and Chiappetta, 2011; Lee *et al.*, 2013). Among them, auxin is a major cell fate-instructive hormone that is synthesized at the shoot tip and transported down to the QC and CSCs at the root tip to form an auxin maximum (Friml *et al.*, 2003; Blilou *et al.*, 2005). The auxin maximum specifies the hypophysis and the QC and regulates formation of the root meristem (Sabatini *et al.*, 1999). The presence of the auxin maximum and the auxin gradient along the root are the result of collective activities and topologies of auxin carrier proteins including the pin-formed (PINs) (Grieneisen *et al.*, 2007). Antagonistic regulation of PIN phosphorylation/dephosphorylation by the pinoid (PID) protein kinase and protein phosphatase 2A (PP2A), respectively, directs topological patterning of PINs and the subsequent auxin flux (Michniewicz *et al.*, 2007).

Brassinosteroids (BRs) are polyhydroxylated steroid hormones that play pivotal roles in a wide range of plant growth and developmental processes (Zhu *et al.*, 2013). Upon binding of a BR to its receptor, brassinosteroid insensitive 1 (BRI1) forms a heterodimeric complex with bri1-associated receptor kinase1/somatic embryogenesis receptor kinase3 (BAK1/SERK3)(Bücherl *et al.*, 2013), and the fully activated BRI1/BAK1 complex then initiates a signalling cascade that activates the positively acting transcription factors brassinazole resistant 1/BRI1 EMS suppressor 2 (BZR1/BES2) and BZR2/BES1 to regulate expression of a wide range of genes and the subsequent plant growth and development (He *et al.*, 2005; Sun *et al.*, 2010; Yu *et al.*, 2011). BR-induced nuclear localization of BZR1/BES1 is crucial for BR activities (Wang *et al.*, 2002; Yin *et al.*, 2002), and this localization is dependent on the phosphorylation and 14-3-3 modification status of the BZR1/BES1 proteins (Gampala *et al.*, 2007; Ryu *et al.*, 2007; Ryu *et al.*, 2010). Brassinosteroid-insensitive 2 (BIN2) is a GSK3/Shaggy-like kinase that phosphorylates BZR1/BES1, thus acting as a negative regulator of the BR signal transduction pathway (Li and Nam, 2002; Yan *et al.*, 2009). In contrast, PP2A dephosphorylates BZR1 and abolishes BZR1 binding to the 14-3-3- proteins to activate BR-responsive gene expression and plant growth (Tang *et al.*, 2011).

BRs regulate root growth in a concentration-dependent manner: they promote root growth at low concentrations and inhibit it at high concentrations (Müssig *et al.*, 2003; Kim *et al.*, 2007). They also play a regulatory role in the control of cell cycle progression and differentiation in the *Arabidopsis* root meristem: plants treated with BR or mutants with enhanced BR signalling, such as *bes1-D*, show premature cell cycle exit that results in early differentiation of meristematic cells (Gonzalez-Garcia *et al.*, 2011). In addition, BRs promote QC division and differentiation of distal stem cells. In fact, the BRI1-like 3 (BRL3) signalosome complex containing BAK1 and BRL1 modulates root growth and development by contributing to the cellular activities of provascular and QC cells (Fàbregas *et al.*, 2013). Recently two BR transcriptional targets— brassinosteroids at vascular and organizing centre (BRAVO) and ethylene response factor 115 (ERF115)—regulating QC quiescence in opposite ways have been identified (Heyman *et al.*, 2013; Vilarrasa-Blasi *et al.*, 2014). BRAVO acts as a cell-specific repressor of QC divisions, and BR-regulated BES1 counteracts its action by directly repressing and physically interacting with the BRAVO (Vilarrasa-Blasi *et al.*, 2014). ERF115 induces QC cell division by acting through phytosulfokines 5 (PSK5) signalling, and BR drives QC proliferation by stimulating *ERF115* expression (Heyman *et al.*, 2013).

Here it is revealed that BR-induced accumulation of BZR1 in the root tips and down-regulation/re-localization of PIN3, PIN4, and PIN7 proteins may result in changes in auxin distribution and the resulting auxin maximum area. This alteration in the auxin localization domain together with gene expression regulation of *BRAVO*, *ERF115*, and other root-regulating genes may lead to ectopic activation of QC division and suppression of CSC differentiation. It is also demonstrated that BR and the related BZR1-/BES1-mediated signalling pathways have opposite effects on the differentiation of distal CSCs in *Arabidopsis* roots in a BR concentration-dependent and a signalling molecule-dependent manner.

## Materials and methods

### Plant materials and growth conditions

Wild-type *Arabidopsis thaliana* (Columbia-0, Col-0), its ethylene biosynthesis mutant *eto1-11* and BR-related mutants (*det2*, *bri1-116*, *bzr1-D*, and *bri1-116*/*bzr1-D*), and wild-type *Arabidopsis thaliana* (Enkheim-2, En-2) and its mutant *bes1-D* plants were used for QC, CSC, and columella cell (CC) phenotype analysis and root-regulating gene expression analysis. *pBZR1::BZR1-YFP* and *p35S::BES1-GFP* plants were used for BZR1 subcellular localization study and ChIP-qPCR assays. Promoter-driven reporter seeds were kindly provided by Dr Wang at Carnegie Institution for Science, USA (*pBZR1::BZR1-YFP*, *pBZR1::bzr1-D-CFP*, and *35S::BES1-GFP*); Dr Laux at the University of Freiburg, Germany (*pWOX5::GFP*); Dr Benfey at Duke University, USA (*pSCR::SCR-GFP*); Dr Guilfoyle at University of Missouri, Columbia, USA (*DR5::GFP* and *DR5::GUS*); Dr Friml at Ghent University, Belgium (*pPIN3::PIN3-GFP*, *pPIN4::PIN4-GFP*, and *pPIN7::PIN7-GFP*); and Dr Scheres at Ultrecht University, The Netherlands (*pCo2::YFP*_*H2B*_, and *J2341*). The following plants were generated: *bzr1-D* x *pWOX5::GFP*, *bzr1-D* x *pSCR::SCR-GFP*, *bzr1-D* x *pC*_*O*_ *2::YFP*_*H2B*_, *DR5::GFP* x *bzr1-D*, and *DR5::GFP* x *mx3* (*pBZR1::bzr1-D-CFP*). These reporter seedlings were used to trace expression and behaviour of the fusion proteins under the confocal laser-scanning microscope.

Seeds were surface-sterilized by sequential washing with 70% EtOH/0.1% Triton X-100 for 20min, 70% EtOH for 10min, and 95% EtOH for 10min. Seeds were dried on a clean bench and cold-treated in the dark at 4 °C for 72h. These sterilized seeds were then sown on 1/2 MS agar medium containing 0.8% phytoagar (pH 5.7) in the presence or absence of the indicated chemicals. Plants were grown in a growth chamber operating under a cycle of 16h light and 8h dark at 23–25 °C with 70% humidity. All seedlings were grown vertically.

### Chemicals

Brassinolide (BL) and X-GLUC were purchased from Duchefa (The Netherlands) and MB Cell (USA), respectively. All other chemicals used in this report were purchased from Sigma-Aldrich (USA) unless otherwise indicated. Brassinazole (BRZ), bikinin (BK), and α-(phenyl ethyl-2-one)-IAA (PEO-IAA) were kindly provided by Dr Yoshida (RIKEN, Japan), Dr Beeckman (Ghent University, Belgium), and Dr Hayashi (Okayama University of Science, Japan), respectively.

### Histochemical staining

**Fig. 1.**
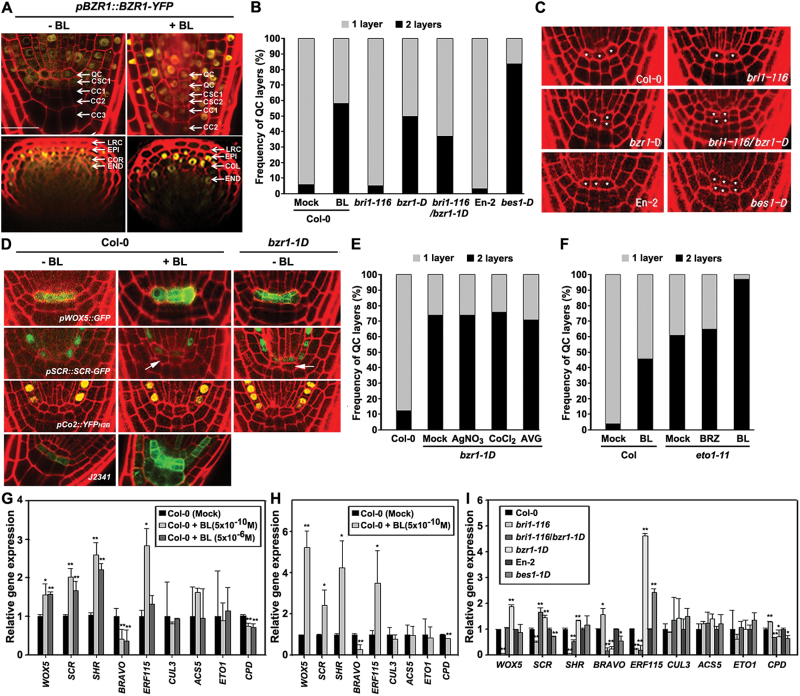
Mitotic reactivation of the QC promoted by BZR1-/BES1-mediated BR signalling pathways and their independency of ethylene-induced QC reactivation. (A) BL-induced nuclear localization of BZR1 in the QC, CSC, and CC cells. Images show YFP expression of representative seedlings in longitudinal (upper panel) and radial (lower panel) cross sections. LRC, lateral root cap; EPI, epidermis; COR, cortex; END, endodermis. *n*>20 seedlings for each treatment. Scale bar, 20 μm. (B) Effects of BL and BR-related genetic background on the frequency of QC cell division. (C) Pictures of representative seedlings used for the cell layer measurements presented in (B). The asterisks (*) represent the position of the QC, determined by morphologic observation. Images show root tips of representative seedlings. (D) Effects of BL and *bzr1-D* genetic background on the expression of root cell-type markers. The arrows indicate the QC where expression of *SCR* marker gene is not detected. *n*>40 seedlings for each treatment. Images show representative reporter expression. All seedlings in (A–D) were grown on 1/2 MS agar media for 7 DAG in the presence (10^−10^ M) or absence of BL. (E) Effects of ethylene biosynthesis or signalling inhibitors on *bzr1-D*-mediated QC cell division. Plants were grown on 1/2 MS media for 8 DAG in the presence of absence of the indicated chemicals. AgNO_3_ (10 μM), CoCl_2_ (200 μM), AVG (1 μM). *n*>50 seedlings for each treatment. (F) Effects of BL or a BL-biosynthetic inhibitor on ethylene overproducer, *eto1-11*. Plants were grown on 1/2 MS media for 6 DAG in the presence of absence of the indicated chemicals. BL (10^−10^ M), BRZ (10^−6^ M). *n*>50 seedlings for each treatment. (G–I) Quantitative RT-PCR analysis of the QC-regulating genes in BL-treated Col-0 and BR-related mutant plants. Col-0 plants were grown on 1/2 MS agar media for 7 DAG and their seedlings were treated with liquid BL for 3h to evaluate short-term effects of BL treatment (G). For evaluation of BL’s long-term effects, Col-0 plants were grown on 1/2 MS agar media for 7 DAG in the presence or absence of BL (H). To analyse expression of QC-related genes in diverse BR mutant plants, Col-0 or En-2 and their mutant plants were grown on 1/2 MS agar media for 8 DAG (I). Values in the graph represent mean expression of genes ± standard deviation, relative to untreated Col-0 control (G and H) or Col-0 or En-2 control (I), which was set as 1.0. The asterisk indicates statistical difference from the mock control (G and H) or from Col-0 or En-2 control (I) at *P*<0.05(*) or at *P*<0.01(**). All qRT-PCR analysis was repeated with a minimum of triple biological replicates and the data were statistically analysed by the Student’s *t*-test. *CPD* was used as a control for BR-repressed genes. (This figure is available in colour at *JXB* online.)

For propidium iodide (PI) staining (reported in Figs 1 and 5), roots were incubated for 30min in PI (5 μg/ml), rinsed, and mounted in H_2_O before confocal microscopic observation.

Staining and detection of β-glucuronidase (GUS) activity was performed according to the method of Jefferson (1987), with some modifications. In brief, roots were fixed in 90% acetone for 20min and infiltrated with staining buffer [0.1M phosphate (pH 7.0), 10mM EDTA, 2.5mM K_4_Fe(CN)_6_·3H_2_0, and 1mg/ml X- GLUC]. The resulting stained root tissues were then fixed and processed as described in mPS-PI staining method.

**Fig. 2.**
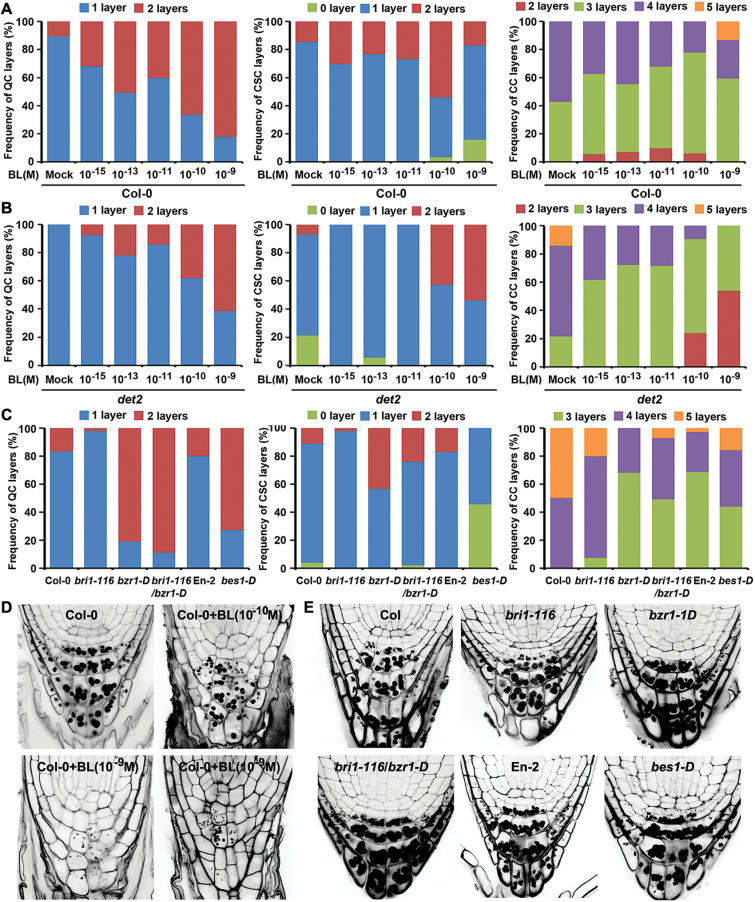
BL dose-dependent and BZR1-/BES1-mediated signalling pathways have differential effects on the differentiation of distal CSCs. (A–B) Concentration-dependent effects of BL on the QC and the distal meristem differentiation in Col-0 (A) and *det2* (B) plants. (C) Effects of BR-related genetic background on the QC and distal meristem differentiation. (D) Pictures of representative seedlings used for the cell layer measurements presented in (A). Note that some Col-0 plants treated with higher concentrations of BL (10^−9^ M) have no CSC. (E) Pictures of representative seedlings used for the cell layer measurement presented in (C). Seedlings were grown on 1/2 MS agar media for 7 DAG in the presence or absence of the indicated concentration of BL (A) or 1/2 MS agar media for 8 DAG (C) before measurement of the QC, CSC, and CC layer frequencies. mPS-PI staining was performed to reveal cell shape, the presence of starch granules. Cells with PI-stained starch granules (black dots) or cells below the starch-stained cell layers were considered columella cells. Cells between QC layers and the starch-stained CC layers were considered CSC cell layers. *n*>50 seedlings for each treatment. (This figure is available in colour at *JXB* online.)

**Fig. 3.**
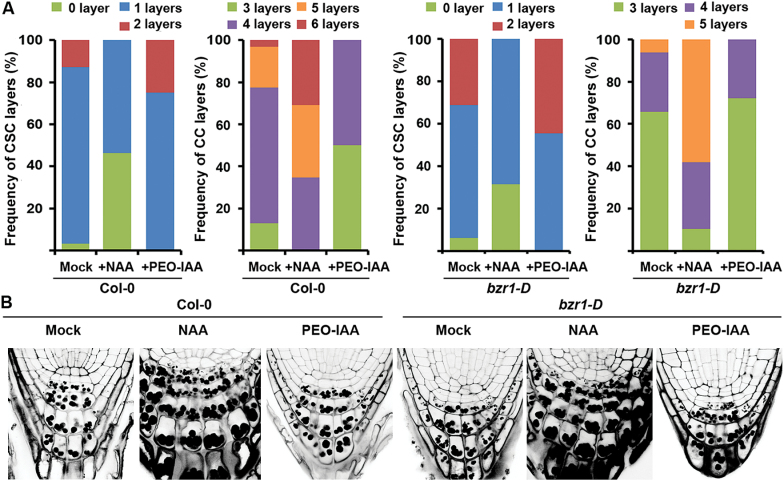
BZR1-mediated BR signalling pathway partly acts upstream of auxin in the regulation of CSC differentiation. (A) Effects of auxin-related chemicals on the differentiation of distal CSCs in Col-0 and *bzr1-D* plants. (B) Pictures of representative seedlings used for the cell layer measurement presented in (A). Seedlings were grown for 8 DAG on 1/2 MS agar media in the absence or presence of NAA (10^−6^ M) and PEO-IAA (2×10^−5^ M). The CSC and CC layers were identified as described in Fig. 2. *n*>50 seedlings for each treatment. (This figure is available in colour at *JXB* online.)

**Fig. 4.**
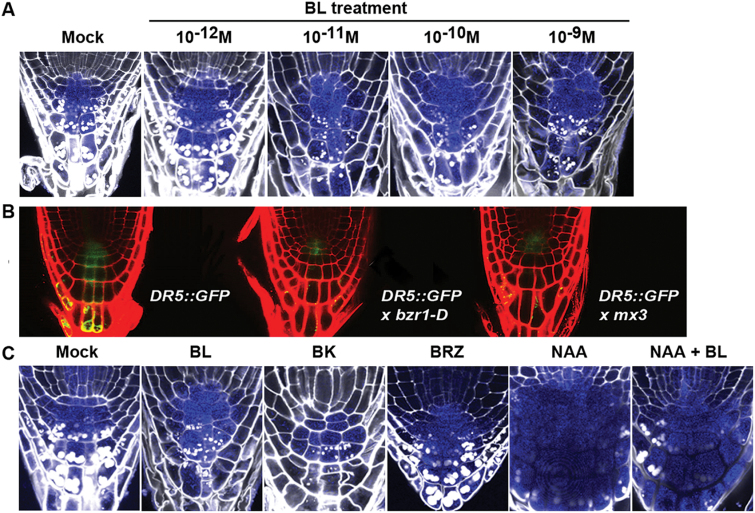
Proximal relocation of the auxin maximum resulting from BZR1-mediated BR signalling pathway in root tips. (A–B) BL concentration-dependent (A) and *bzr1-D*-dependent (B) proximal relocation of the auxin-localizing domain. (C) Effects of BL- and auxin-related chemicals on establishment of the auxin localization domain. Seedlings expressing an auxin reporter (*DR5::GUS* or *DR5::GFP*) were grown on 1/2 MS agar media for 7 DAG in the presence or absence of the indicated concentration of BL (A), or with BL (10^−10^ M), BK (3×10^−5^ M), BRZ (10^−6^ M), NAA (10^−6^ M), and NAA (10^−6^ M) + BL (10^−10^ M) (C). The photographs show representative seedlings for each treatment. White dots, starch granules; bluish area, auxin-accumulating area. *n*>40 seedlings for each treatment. (This figure is available in colour at *JXB* online.)

mPS-PI staining was performed according to the method of Truernit *et al.* (2008) with some modifications to reveal cell shape, the presence of starch granules, and auxin maximum (described in Figs 2, 3 and 4). In brief, roots were fixed in fixative (50% methanol and 10% acetic acid) and then incubated in 1% periodic acid after rinsing with water. Next, the tissues were treated with modified Pseudo Schiff reagent (100mM sodium metabisulphite and 0.15N HCl, supplemented with freshly added PI to a final concentration of 100 μg/ml). The samples were transferred onto microscope slides and covered with a chloral hydrate solution (4g chloral hydrate, 1ml glycerol, and 2ml water) for 5min to observe fluorescence or reflection signals using the Zeiss Confocal microscope.

### Confocal microscopy

PI, YFP/GFP, and GUS signals were obtained using a Zeiss LSM 710 confocal laser-scanning microscope equipped with an argon 2 ion laser (488nm) and a He/Ne 1 ion laser (543nm). For observation of PI-stained roots, samples were excited at 543nm to collect the PI signal at 595–615nm or excited at 488nm to detect GFP signal at 493–545nm or YFP signal at 521–560nm. For observation of mPS-PI-stained roots, samples were excited at 488nm and the emission was collected at 520–720nm to detect the PI signal. The stained signal for GUS activity was obtained using the reflection mode of the confocal microscope. In this case, the excitation wavelength was 488nm, and the reflection signal was collected at 485–491nm. Images were compiled and analysed using the LSM 710 ZEN image browser software.

### Quantitative real-time PCR analysis

**Fig. 5.**
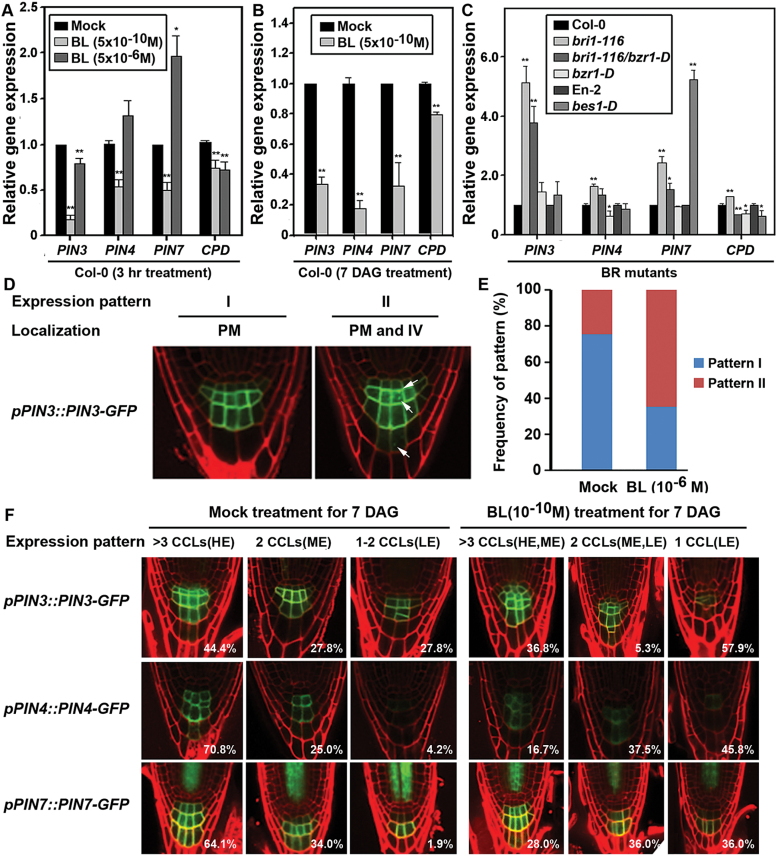
BL regulates expression of *PIN* genes in a concentration- and BZR1-dependent manner and induces changes in expression domain and subcellular localization of internalization of PIN proteins. (A–C) Quantitative RT-PCR analysis of *PIN* genes in BL-treated Col-0 and BR-related mutant plants. Plants were grown and *PIN* gene expressions were analysed for short-term BL-treated plants (A), long-term BL-treated plants (B), and BL-related mutants (C) as described in Fig. 1G–I. (D–E) Short-term effect of BL on subcellular localization of PIN3-GFP protein. The PIN3 reporter plants were grown on 1/2 MS agar media for 7 DAG and treated with BL (10^−6^ M) for 3h in solution. The images in (D) show GFP expression of representative seedlings for each PIN3 expression pattern of I and II. The graph in (E) presents the frequency of plants with either expression pattern I or II in mock- and BL-treated seedlings. *n*>50 seedlings for each treatment. PM, plasma membrane; IV, intracellular vesicles. (F) Long-term effect of BL on PINs-GFP protein expression. The PIN reporter seedlings were grown on 1/2 MS agar media for 7 DAG in the presence or absence of BL (10^−10^ M). Expression pattern indicates number of expressing columella cell layers (CCL) and intensity (HE, high expression; ME, medium expression; LE, low expression) of the corresponding PINs-GFP protein. Numbers in each picture represent the frequency of plants with the corresponding expression pattern in the mock- or BL-treated reporter plants. *n*>60 seedlings for each treatment. (This figure is available in colour at *JXB* online.)

**Fig. 6.**
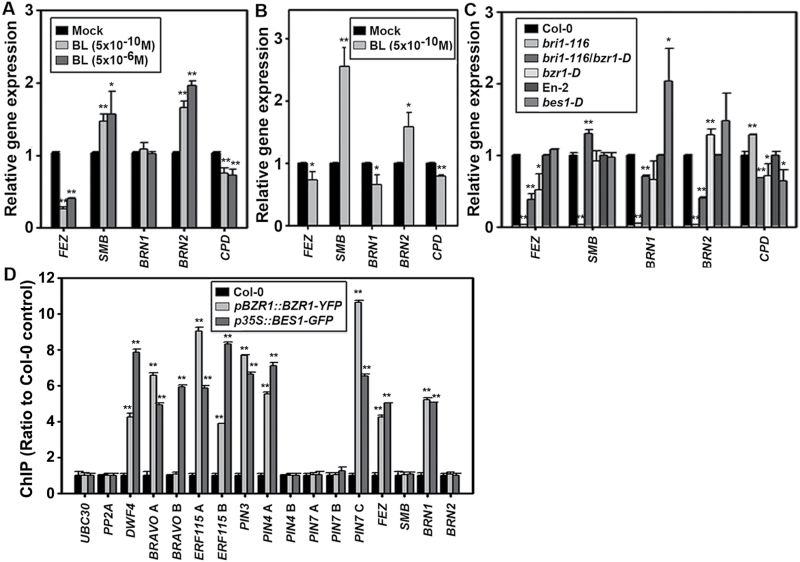
BR- and BZR1-mediated signalling pathway modulates transcriptional expression of diverse root-regulating genes. (A–C) Quantitative RT-PCR analysis of root-controlling genes in BL-treated Col-0 and BR-related mutant plants. Plants were grown and expression of root-regulating genes was analysed for short-term BL-treated plants (A), long-term BL-treated plants (B), and BL-related mutants (C) as described in Fig. 1G–I. (D) ChIP-qPCR assay of the BZR1- and BES1- binding *cis*-motif of root-regulating genes. BZR1- and BES1-bound chromatin DNA fragments were isolated from roots of week-old Col-0, *pBZR1::BZR1-YFP*, and *p35S::BES1-GFP* plants using anti-BZR1 antibodies. Next, quantitative PCR analysis was performed with the primer sets listed in Supplementary Table S2 as described in Materials and methods. Each primer set amplifies a region of the promoter simplified in Supplementary Figure S2 (available at *JXB* online) containing the BZR1-binding BRRE sequence (5′-CGTGT/CG-3′, He *et al.*, 2005) and E-box (5′-CANNTG-3′, Yin *et al.*, 2005). Promoters of *UBC30* and *PP2A* genes were used as negative controls for BZR1- and BES1-binding motif. The promoter of *DWF4* was used as a positive control (Sun *et al.*, 2010). All ChIP-qPCR analysis was repeated with a minimum of triple biological replicates and the data were statistically analysed by the Student’s *t*-test.

Total RNA was isolated from root tissues using the Plant RNA Extraction Kit (Intron Biotechnology, Korea) and treated with RQ1 RNase-Free DNase (Promega, USA) according to the manufacturer’s instructions. Quantitative real-time PCR (qRT-PCR) was performed by the SYBR green method using the Applied Biosystems Step One Plus System (Applied Biosystems, USA). The expression of each transcript was normalized against the amount of *UBC1* control in each sample. *CPD* was used as a control for BR-repressed genes. The results were reported as expression relative to the Col-0 mock control (Figs 1G, 1H, 5A, 5B, 6A, and 6B) or Col-0 or En-2 genetic control (Figs 1I, 5C, and 6C). Three to five biological replicates were included in each experiment, and the data were statistically analysed using the Student’s *t*-test. Primers used for the qRT-PCRs are summarized in Supplementary Table S1 (available at *JXB* online).

### ChIP-qPCR assay

ChIP experiments were performed as previously described with minor modifications (Gendrel *et al.*, 2005) using the non-transgenic Columbia-0, plants expressing native promoter-driven BZR1-GFP (*pBZR1::BZR1-YFP*), and plants constitutively expressing BES1-GFP (*p35S::BES1-GFP*). In brief, week-old seedlings were cross-linked with 1% formaldehyde and the chromatin DNA fragments (~500bp in length) were isolated from their nuclei of root tissues to retrieve BZR1 or BES1-binding DNA fragments using rabbit polyclonal anti-GFP antibodies (Abcam, UK). The bound DNA fragments were then extracted using the Gel/PCR DNA fragments extraction kit (RBC Bioscience, Korea) and the amount of the bound DNA was measured by performing real-time PCRs with the primer sets listed in Supplementary Table S2 (available at *JXB* online) using equal amounts of DNA from the input and the CoIP fractions.

## Results

### Both BZR1- and BES1-mediated BR signalling pathways stimulate mitotic reactivation of QC cells

The QC is the source of stem cell initials and is maintained at the G1/S cell cycle checkpoint and thus divides infrequently (Jiang and Feldman, 2005). BR-induced nuclear localization of BES1, a positive regulator of the BR signalling pathways, in the QC promotes QC reactivation, leading to production of two layers of QC cells (Gonzalez-Garcia *et al.*, 2011; Heyman *et al.*, 2013; Vilarrasa-Blasi *et al.*, 2014) and results in CSC differentiation (Gonzalez-Garcia *et al.*, 2011). In this report, whether BZR1, a BES1 homologue, is also actively involved in BR-regulated QC regulation was tested. BZR1 is primarily localized in the nucleus of epidermal cells and some cortex cells of the proximal root tips of *pBZR1:BZR1-YFP* plants when they are grown in medium lacking BL (a type of BR) (Fig. 1A). It was found that, contrary to a previous report (Vilarrasa-Blasi *et al.*, 2014), supplying BL in the medium enhanced nuclear localization of BZR1 in the cells of the SCN area, so that concentrated nuclear expression was found in the root tip cells including the QC, cells above the QC, the CSC, and CCs (Fig. 1A). Similar localization of BZR1 was evident within 15min when BL was applied to 7-d-old seedlings (data not shown). This implies that not only the reported BES1- but also the BZR1-mediated BR signalling pathway may actively be involved in QC division and the distal CSC differentiation.

To assess roles of the BZR1-mediated signalling pathway in these developmental processes, whether the BR and BZR1-mediated signalling pathway modulates the mitotically inactive status of the QC was investigated. Col-0 plants grown on medium containing 10^−10^ M BL showed an increased frequency of mitotically activated QC cells, such that 58% of plants had two layers of QC cells with periclinal division by 7 d after germination (DAG) as determined by morphologic lineage (Fig. 1B). In comparison, 5% of untreated Col-0 plants harboured double layers of QC cells. Consistent with a previous report (Gonzalez-Garcia *et al.*, 2011), more than 80% of plants with the *bes1-D* BR-signalling enhancing mutation in an En-2 background showed reactivated QC cell layers by 7 DAG, increasing to 100% by 14 DAG (Fig 1B, C; Supplementary Table S3, available at *JXB* online). It was next examined whether BZR1-mediated BR signalling results in similar QC reactivation. It was found that the QC was also frequently reactivated in BR-signalling enhanced *bzr1-D* plants, such that two QC layers were observed in 50% of the plants by 7 DAG and in 75% of the plants by 14 DAG (Fig. 1B, C; Supplementary Table S3). This QC reactivation was less frequent than that in *bes1-D* plants, but was greatly enhanced compared with Col-0 and BR-signalling defective *bri1-116* plants, which hardly ever showed QC division at 7 DAG. Introduction of *bzr1-D* clearly reactivated the QC of *bri1-116* (divided QCs were present in 37% of the *bri1-116*/*bzr1-D* plants by 7 DAG and 69% by 14 DAG), which implies that BRI1/BZR1-mediated BR signalling is actively involved in reactivation of QC division.

*WOX5* expression in the embryonic cell lineage leads to QC formation and is involved in maintaining the stem cell state in a non-autonomous manner (Haeker *et al.*, 2004; Sarkar *et al.*, 2007). It was demonstrated that the QC marker *WOX5* was expressed in both divided QC cells while a cortex marker (*pC*_*O*_ *2*::*YFP*_*H2B*_, Heidstra *et al.*, 2004), was unchanged in its expression pattern when these plants were grown on medium containing BL (Fig. 1D). SCARECROW (SCR) is required for specification of QC identity, stem cell homeostasis and asymmetric cell division (Sabatini *et al.*, 2003; Moubayidin *et al.*, 2013). In this study, the SCR reporter plants grown on medium containing BL reactivated QC cell division as determined by morphologic cell lineage but, unexpectedly, SCR protein accumulated predominantly in the upper QC in most BL-treated cells. To verify the effect of BR on QC division, *bzr1-D* was crossed with different cell-specific marker lines (Fig. 1D). For *bzr1-D*×*pWOX5::GFP*, 50% of the plants showed GFP expression in both the upper and lower cells of the QC whereas the other 50% showed expression only in the upper cell. In most *bzr1-D*×*pSCR::GFP*, the GFP signal was present only in the upper cell of the QC. This lack of *WOX5* and *SCR* expression in the rootward QC cells may indicate an asymmetric QC division and loss of functional QC identity.

Ethylene overexpressor 1 (ETO1) and CULLIN3 (CUL3) are a part of the ubiquitin E3 ligase complex that modulates the level of 1-Aminocyclopropane-1 carboxylic acid (ACC) synthase 5 (ACS5) catalysing the rate-limiting step of ethylene biosynthesis. It was previously reported that the gain-of-function *eto1-11* mutant overproduces ethylene and promotes QC cell division (Ortega-Martinez *et al.*, 2007). In this regard, it was tested whether the BZR1-mediated BR signalling pathway interacts with ETO1-promoted ethylene pathway in the regulation of the QC cell division. *bzr1-D* plants grown for 8 DAG showed QC cell division at a frequency of 74% (Fig. 1E). Treatment of neither an ethylene action inhibitor AgNO_3_ nor biosynthetic inhibitors, aminoethoxy-vinylglycine (AVG) and CoCl_2_, interfered with this *bzr1-D*-induced QC cell division. Similarly, treatment of *eto1*-*11* mutants with BRZ, a BR biosynthesis inhibitor (Asami *et al.*, 2000), did not significantly influence the QC reactivation frequency of *eto1*-*11* plants (Fig. 1F). Moreover, both *eto1*-*11* genetic background provoking QC cell division at a frequency of 61% and treatment of BL inducing cell division at a frequency of 45% additively coordinated the promotion of QC cell reactivation, thus BL-treated *eto1*-*11* plants showed two-layered reactivated QC cells at a frequency of 97%. These results indicate that BZR1-mediated BR signalling and ETO1-promoted ethylene signalling pathways independently act on the QC reactivation event in a parallel manner.

Supporting BZR1-mediated QC reactivation, expression of *WOX5*, *SCR*, and *SHR* genes was significantly increased by both short-term (3h, Fig. 1G) and long-term (7 DAG, Fig. 1H) treatments of BL. Consistent with BR-induced up-regulation, their expression was significantly repressed in BR signalling-defective *bri1-116* plants, but greatly increased in signalling-enhanced *bzr1-D* plants and *bri1-116*/*bzr1-D* double mutants (Fig. 1I). In addition, BL treatment greatly suppressed gene expression of *BRAVO* (a negative regulator of QC divisions; Vilarrasa-Blasi *et al.*, 2014) and increased the expression of *ERF115* (a positive regulator of QC division; Heyman *et al.*, 2013) in both the short-term and long-term treated Col-0 plants (Fig. 1G, H). Again, their expression was tightly controlled in a BRI1/BZR1-mediated signalling-dependent manner (Fig. 1I). Expression of *BRAVO* and *ERF115* was similarly regulated in *bes1-D* plants to those of *bzr1-D* plants. Expression of genes regulating ethylene biosynthesis was also examined, and it was found that expression of *CUL3*, *ACS5*, and *ETO1* was not regulated by BR treatment nor was it different in *bzr1-D* or *bes1-D* plants compared with the Col-0 or En-2 plants, respectively (Fig 1G–I). These results support the conclusion that BRs promote QC cell division independently from the ethylene pathway.

### BRs apparently exert opposite effects on the differentiation of distal CSCs in a concentration- and BZR1-/BES1-dependent manner

Results presented thus far have shown that the BRI1/BZR1-mediated BR signalling pathway was able to reactivate and produce multiple layers of the QC (Fig. 1B; Supplementary Table S3 available at *JXB* online). In addition, BL treatment greatly enhanced expression of a columella initial marker, *J2341*, and expanded its expression to the QC, CC, and other root tip cells (Fig. 1D). These observations, together with BL-induced intense nuclear localization of BZR1 in the QC, CSCs, and CCs (Fig. 1A), prompted an investigation of the effects of BR on the maintenance and differentiation of distal CSCs by examining the development of starch-accumulated columella cells.

First, the cell layer number of QC, CSC, and CC was measured for Col-0 (Fig. 2A) and BR biosynthesis-defective *de-etiolated2* (*det2*; Noguchi *et al.*, 1999) (Fig. 2B) plants grown on media containing various concentrations of BL, ranging from 10^−15^ to 10^−9^ M. To do this, root tips were stained with PI by performing mPS-PI staining to mark cell walls and starch granules. As expected, BL stimulated division of the QC in a concentration-dependent manner in both Col-0 and *det2* plants. Less than 10% of Col-0 plants grown on media lacking BL carried two layers of QC, and the proportion of plants with the divided QC was greatly increased after exogenous treatment with BL: 60% and 82% of Col-0 plants grown on medium containing 10^−10^ M and 10^−9^ M, respectively, contained two layers of QC (Fig. 2A, D). In contrast, differentiation of CSC into CC was inhibited by the BL treatment. Most Col-0 plants grown on media lacking BL carried a single layer of CSCs, and only 15% of plants had two layers of CSCs. The proportion of Col plants with two CSC layers was moderately increased (25–30%) by the 10^−15^ M to 10^−11^ M of BL treatment, and it reached to 52% by the 10^−10^ M BL treatment, which implies that BL inhibits CSC differentiation into CC. Consistent with this, treatment of exogenous BL reduced the number of CC layers. For example, Col plants grown without exogenous BL had 57% seedlings with four layers of CCs and 43% with three layers of CCs (Fig. 2A). Interestingly, the proportion of plants with fewer CC layers steadily increased with increasing amount of BL treatment, so that plants grown on medium containing 10^−10^ M BL showed 25% seedlings with four layers of CCs, 68% with three layers of CCs, and 7% with two layers of CCs. These concentration-dependent developmental trends of QC, CSC, and CC layers were clearly reproduced in BL-treated *det2* plants (Fig. 2B).

Surprisingly, treatment of Col-0 plants with a higher concentration of exogenous BL resulted in opposite effects on CSC differentiation and CC development: it promoted CSC differentiation and the resulting CC development. Seventeen percent of Col-0 plants grown on 10^−9^ M BL had two layers of CSCs, which is far below the frequency compared with those of the plants grown on 10^−15^ M to 10^−11^ M of BL, and is about the same frequency as with mock-treated plants. Moreover, plants with no CSC (whose starch granules of cells resided underneath the QC were stained with PI) were present at a frequency of 18%, which was hardly observed in the mock- and lower BL-treated Col-0 plants (Fig. 2A, D). Consistent with this observation, an increased frequency of plants with a higher number of CC layers was observed in plants grown on medium containing 10^−9^ M BL than in plants grown with lower concentrations of BL. Similar observations were previously reported in Col-0 plants treated with 4×10^−9^ M BL and the *bes1-D* BR gain-of-function mutant (Gonzalez-Garcia *et al.*, 2011). Interestingly, this effect was not observed in BL-treated *det2* plants (Fig. 2B). Treatment of BR-deficient *det2* plants with higher concentrations of BL further increased the number of CSC layers and dramatically decreased the number of CC layers accordingly. These results strongly indicate that BRs differentially regulate distal CSC differentiation in a concentration-dependent manner: they inhibit the stem cell differentiation at low concentrations and promote it at higher concentrations. In support of data presented here, Col-0 plants grown on media containing BK, an inhibitor of BIN2 (a negative regulator of the BR signalling pathway) (De Rybel *et al,* 2009) showed similar results to plants treated with lower concentrations of BL (data not shown). Also of note was that BL treatments not only modulated CSC differentiation, as determined by counting the number of cell layers stained with PI or below the stained cells, but also weakened the intensity of PI staining (Fig. 2D). At the extreme, most Col-0 plants treated with 10^−9^ M BL had 1–3 cells in the lowest layers that were not stained at all. This result implies that BR may be involved in starch development as well as differentiation of the distal meristem.

Next, to investigate the BRI1/BZR1-dependent regulation of QC division and CSC differentiation, the frequency of QC, CSC, and CC layers in diverse BR-related mutant plants was examined. As previously reported (Gonzalez-Garcia *et al.*, 2011), *bes1-D* plants grown in MS media under the present experimental conditions showed enhanced QC reactivation and distal cell differentiation (Fig. 2C, E). Twenty percent of En-2 plants had two layers of QC and the remaining 80% carried a single QC cell layer. In comparison, more than 70% of *bes1-D* plants harboured two-layered QCs. Moreover, the proportion of *bes1-D* plants with no CSC also dramatically increased. It was found that 52% of the *bes1-D* plants had single-layered CSCs and the rest had no CSC, while 83% of En-2 plants carried single-layered CSCs and the remaining 17% had two-layered CSCs. In accordance, the frequency of plants with higher CC layers was increased in the *bes1-D* in that 16% of the plants had five layers, 39% four layers, and 45% three layers. In comparison, En-2 plants showed 3% with five-layered, 27% with four-layered, and the remaining 70% with three-layered CCs.

Comparing Col-0 plants with *bri1-116*, *bzr1-D*, and *bri1-116*/*bzr1-D* plants, 17% of Col-0 plants had two-layered QCs and the remaining 83% showed single-layered QCs by 8 DAG on MS media (Fig. 2C, E). In the same population of Col-0 plants, it was found that 90% of plants carried single-layered CSCs and a half developed four-layered CCs while the other half developed five-layered CCs. In comparison, the frequency of plants with single-layered QCs and CSCs in a BR-signalling defective mutant was slightly increased so that 98% of the *bri1*-*116* plants carried single-layered QC or CSC. This implies that a defective BR signalling pathway may result in retardation of QC or CSC cell division or differentiation. Development of CCs was also slightly inhibited compared with the Col-0 plants: 20% of *bri1*-*116* plants had five-layered, 72% four-layered, and the remaining 8% had three-layered CCs. Interestingly, a similar trend of reduction in CC layers was also observed in the BRZ-treated Col plants (data not shown). A BR-signalling enhanced mutant, *bzr1-D*, showed an increased frequency of plants with two-layered QCs as expected (80% with two-layered and the remaining 20% with single-layered plants). In addition, the frequency of plants with two-layered CSCs also dramatically increased (45% with two-layered and the rest with single-layered plants) as seen in the case of BL-treated Col-0 plants. Concurrently, the frequency of plants with fewer CC layers in the *bzr1-D* mutant was greatly increased in that 32% of the plants had four CC layers and the remaining 68% had three-layered CCs. A double mutant of the BR signal pathway, *bri1-116*/*bzr1-D*, had a similar frequency of plants with QC layers to *bzr1-D* and an intermediate frequency of plants with CSC and CC layers between *bri1-116* and *bzr1-1D*. These results imply that differentiation of CSC and the resulting development of CC are under the control of the BRI1/BZR1-mediated BR signalling pathway, and that the BZR1-mediated BR signalling pathway, in opposition to BES1-mediated signalling, inhibits the differentiation of distal CSCs, thus delaying development of starch-filled columella cells. In addition, it seems that a defect in either BR biosynthesis (in the case of BRZ-treated Col plants) or in the signalling pathway (in the case of *bri1-116*) show similar results to BL treatment and BZR1-mediated signal enhancement.

### BZR1-mediated BR signalling pathway partly acts on the auxin pathway and shows an antagonistic effect on auxin-stimulated CSC differentiation

The auxin signalling pathway that stimulates differentiation of CSCs requires activation of auxin responding factors, ARF10 and ARF16 and subsequent restriction of *WOX5* transcription in the QC (Wang *et al.*, 2005; Ding and Friml, 2010). Based on these findings, whether the inhibitory effect of the BZR1-mediated BR signalling pathway involves interaction with auxin activities in the distal area was examined. Ninety percent of Col-0 plants grown on MS media without exogenous BL carried a single-layered CSC and the remaining 10% of plants had two layers of CSCs (Fig. 3A, B). In the same population, 23% of the Col-0 plants had five- or six-layered, 64% had four-layered, and 13% had three-layered CCs. As reported previously (Ding and Friml, 2010), Col-0 seedlings grown on medium supplemented with an auxin (1-naphthaleneacetic acid, NAA) led to a decrease in CSC layers and a resulting increase in CC layers. For example, the frequency of plants with single-layered CSCs in NAA-treated Col-0 seedlings was greatly decreased to 52% from 90% in the untreated Col-0 plants. Moreover, 48% of the NAA-treated plants showed no CSC at all. Accordingly, the frequency of NAA-treated plants with more CC layers increased significantly: 30% of the auxin-treated plants carried six-layered CCs, 35% with five-layered, and the remaining 35% had four-layered CCs. Conversely, treatment of plants with an auxin action inhibitor (PEO-IAA, Yoshimoto *et al.*, 2012) increased plants with more CSC layers and with fewer CC layers compared with those of Col-0 plants.

It has been shown in BL-treated Col-0 and *bzr1-D* plants that the frequency of plants with a higher number of CSC layers was increased and the number of CC layers was decreased compared with those of Col plants (Figs 2A–C and 3A). However, NAA greatly negated the effects of the *bzr1-D* mutation, so that it led to a dramatic increase in the frequency of plants with fewer CSC layers and subsequent enhanced differentiation of CSC into CC layers, as in the case of Col-0 plants treated with the same chemicals (Fig. 3A, B). For example, 32% of PEO-IAA-treated *bzr1-D* plants carried no CSC layer while only 6% of the untreated plants had no CSC layer. For CC layers, 58% of PEO-IAA-treated *bzr1-D* plants carried five-layered CSCs while only 5% were observed in untreated plants. In addition, treatment of PEO-IAA to the *bzr1-D* plants slightly augmented the *bzr1-D* mutation effect, so that the frequency of plants with more CSC layers and with fewer CC layers was slightly increased compared with the untreated *bzr1-D* plants. For example, 45% of PEO-IAA-treated *bzr1-D* plants carried two-layered CSCs while 31% of the untreated plants had two-layered CSCs. For CC layers, 72% of PEO-IAA-treated *bzr1-D* plants carried four-layered CSCs while this was observed in 66% of the untreated plants. Nonetheless, it was noticed that treatment of NAA or PEO-IAA to the *bzr1-D* plants did not completely lead to frequencies of CSC and CC layers observed in Col-0 plants treated by the same auxin-related chemicals. These results indicate that BZR1-mediated BR regulation on CSC differentiation and the following CC development partly act on the auxin action antagonistically, and a part of BZR1-mediated BR regulation for these developments may act in parallel to auxin.

### BZR1-mediated BR signalling pathway alters the expression/subcellular distribution of PIN proteins resulting in proximal relocation of the auxin localization domain in roots

Auxin is synthesized at the shoot tip and transported down to the QC and columella initials in the root tip where the auxin maximum is formed (Blilou *et al.*, 2005). Disruption of the auxin expression domain leads to defective development of the distal meristem (Friml *et al.*, 2002). Based on the finding that BR acts antagonistically on auxin action in the regulation of CSC differentiation, whether BR modulates the auxin expression domain (auxin maximum) was examined in the root tip using *DR5::GUS* and *DR5::GFP* plants as auxin-sensing reporters. To do this, roots were stained by performing either a GUS activity assay followed by mPS-PI staining to reveal cell walls and starch granules together with auxin-accumulating cells (Fig. 4A, C), or regular PI staining with live tissues to reveal cell walls (Fig. 4B). Auxin gradually accumulated from the QC to the CCs with the highest concentration at the CSC, CC1, and CC2 areas in most plants grown on MS medium without BL (Fig. 4A). About 35% of seedlings also showed auxin maximum at the root tip cells including the QC. Increasing the concentration of BL in the MS medium to 10^−10^ M shifted this expression domain longitudinally upward so that auxin was heavily concentrated at the QC, CSC, CC1, and the proximal area. For instance, more than 80% of BL (10^−10^ M)-treated *DR5::GUS* seedlings showed this pattern of auxin expression. Similarly, the *DR5::GFP* expression domain in *bzr1-D* plants and *mx3* transgenic plants ectopically expressing the mutated form of *BZR1* (*pBZR1::bzr1-D-CFP*) moved to the proximal area (Fig. 4B). Consistent with the BL-driven proximal shift of the auxin maximum, BK treatment mimicking the BR-induced positive signalling pathway moved the auxin-accumulating domain to the proximal area, whereas treatment of the GUS reporter plants with BRZ reversed the BR effect on the maximum movement (Fig. 4C). Interestingly, some *DR5::GUS* plants treated with 10^−9^ M BL resulted in enforced accumulation of auxin in the root tip area including the proximal zone, QC, and CCs (Fig. 4A). This is coincident with the finding that treatment of Col-0 plants with higher concentrations of exogenous BL resulted in opposite effects on CSC differentiation and CC development compared with those induced by lower concentrations of BL (Fig. 2A, D). As expected, the gradual expression of *DR5::GUS* and correct establishment of auxin maximum were dramatically abolished in plants grown in medium supplied with NAA, in which auxin was uniformly accumulated in all root tip areas at a high level (Fig. 4C). Supplying BL together with the NAA in the growth medium did not reverse this tendency, thus a high amount of auxin remained through the root tip.

The presence of the auxin maximum and the auxin gradient along the root are the result of collective activities and topologies of the PIN proteins (Grieneisen *et al.*, 2007). Specifically, PIN3, PIN4, and PIN7 play important roles in the positioning of auxin maximum and the following regulation of root patterning and distal meristem differentiation (Friml *et al.*, 2002; Blilou *et al.*, 2005). To examine whether BR modulates transcript expression and protein localization of PINs in the root tip area, BL was applied to the root of Col-0 and PIN reporter plants for 3h or for 7 DAG and their gene expression and subcellular protein localization was examined. Interestingly, BL differentially regulated the expression of *PIN*s in a concentration-dependent manner: treatment of Col-0 roots for a short period (3h) with BR at low concentration (5×10^−10^ M) greatly suppressed expression of *PIN3*, *PIN4*, and *PIN7* genes whereas treatment with a higher concentration of BL (5×10^−6^ M) resulted in enhanced expression of *PIN7* (Fig. 5A). A similar down-regulation of *PIN* gene expression was also observed for plants growing on a MS solid medium containing 5×10^−10^ M BL for a long period (7 DAG) (Fig. 5B). This regulation of *PIN* genes, especially for *PIN4*, was dependent on BRI1-/BZR1-mediated signalling pathways so that gene expression of *PIN3*, *PIN4*, and *PIN7* was significantly enhanced in a *bri1-116* background compared with the Col-0 plants. On the other hand, expression was significantly down-regulated (*PIN4*), slightly increased (*PIN3*) without statistical significance, or maintained at the Col-0 control level (*PIN7*) in *bzr1-D* plants (Fig. 5C). Moreover, up-regulation of the *PIN* genes by *bri1-116* mutation was partly negated by the *bzr1-D* mutation background in *bri1-116*/*bzr1-D* double mutants. This *bzr1-D* down-regulation effect of *PIN* genes resembles exogenous BL treatment at lower concentrations. In contrast, *PIN7* expression was greatly increased in *bes1-D* mutants, which resembles the gene expression in plants treated with a higher concentration of BL. These results are consistent with the observation that BR has opposing effects on the differentiation of distal CSCs in concentration- and BZR1-/BES1-dependent manners.

Next, the PIN reporter plants were treated with BL, as done for the gene expression analysis, and PIN protein expression and subcellular localization was examined (Fig. 5D–F). Without BL treatment, PIN3-GFP protein of *pPIN3::PIN3-GFP* plants in this experimental condition accumulated in the cell membrane of CSC and CC1 to the CC3 with predominant expression in CC1 and CC2 (Fig. 5D, F; upper panel). In contrast when BL was added to the incubation medium at a higher concentration (10^−6^ M) for 3h, the PIN3 expression domain was restricted mainly to CC1 and CC2 and disappeared from CC3. At the same time, the characteristic plasma membrane-localized PIN3 pattern was gradually diminished, and PIN3 appeared in endosome- or vesicle-like internal structures (Fig. 5D) at a frequency of 25% in mock-treated plants, which jumped to 65% in the BL-treated plants (Fig. 5E). Finally, growing PIN3 reporter seedlings on medium supplemented with 10^−10^ M BL for 7 DAG resulted in a significant decrease in the number of PIN3 expression layers and intensity of the PIN3 protein expressed (Fig. 5F). For example, 44.4% of the reporter plants showed PIN3-GFP expression in >3 columella cell layers with high expression level while 27.8 % expressed in 1–2 columella cell layers with low expression intensity. In contrast, BL-treated plants displayed 36.8% of plants with expression in >3 columella cell layers with high or medium expression intensity, and 57.9% of plants showed expression in one columella cell layer with low expression intensity. PIN4-GFP protein showed a nonpolar localization mainly in the membrane of CC1 to CC3 with prominent basal accumulation (Fig. 5F; middle panels). PIN7-GFP protein showed a similar expression pattern with prominent expression in CC2 (Fig. 5F; lower panel). Similar to the BL-treated *PIN3::PIN3-GFP* plants, treatment of BL (10^−10^ M) to *pPIN4::PIN4-GFP* and *pPIN7::PIN7-GFP* plants for 7 DAG resulted in a reduction in expression layers and intensity of reporter proteins. In conclusion, the BR signalling pathway modulates PIN gene expression and protein localization, which may lead to proximal relocation of the auxin maximum in the root tip.

### BR regulates transcriptional expression of diverse root-controlling genes in a BZR1-dependent manner

Transcriptional regulation of many protein factors plays an important role in the establishment, maintenance, and differentiation of the root apical meristem (reviewed in Lee *et al.*, 2013). In this report, results have shown that BR-induced and BZR1-mediated signalling pathway regulates expression of several root-regulating genes including *PIN*s and *WOX5*. To have a better understanding of how BR regulates CSC differentiation, transcriptional expression of other key root-regulating genes was examined in the presence or absence of BL.

SOMBRERO (SMB) and FEZ proteins antagonistically regulate distal stem cell differentiation through transcriptional negative feedback regulation: FEZ promotes periclinal root cap-forming cell divisions and SMB negatively regulates FEZ activity, repressing stem cell-like divisions in the root cap daughter cells (Willemsen *et al.*, 2008). Therefore, it was determined whether BR regulates expression of *SMB* and *FEZ* and whether the mode of this regulation is opposite for these genes. In Col-0 plants, BL up-regulated *SMB* expression whereas it down-regulated *FEZ* expression for short- and long-treatment with both low and high concentrations of BL (Fig. 6A, B). In *bri1-116* plants, *SMB* expression was down-regulated but recovered to the level of Col-0 plants by introducing a *bzr1-D* mutation (*bri1-116*/*bzr1-D*, Fig. 6C). Given the fact that the *smb-3* mutant has more CC layers and *fez-2* has fewer CC layers (Willemsen *et al.*, 2008), these results indicate that the BR-induced up-regulation of *SMB* and down-regulation of *FEZ* may contribute to the BR-induced and BZR1-mediated suppression of CSC differentiation. In support of this idea, *FEZ* expression was significantly down-regulated in *bzr1-D* plants. BEARSKIN 1 (BRN1) and BRN2, together with SMB, directly activate transcription of both vascular nac domain (*VND*) and nac secondary wall thickening promoting factor (*NST*) to regulate cell maturation in the root cap, which undergoes terminal differentiation and detachment from the root (Bennett *et al.*, 2010). The results showed that BR up-regulated *BRN2* expression in Col-0 plants during both short- and long-term treatment, and that this was under the control of BRI1/BZR1-mediated gene expression (Fig. 6A–C). The expression of *BRN1* in Col-0 plants was not significantly changed by short-term treatment of BL, but was slightly decreased by prolonged treatment with 5×10^−10^ M of BL. Interestingly, its expression was enhanced in *bes1-D* plants. Further studies are needed to carefully analyse how this regulation of gene expression is linked to BR- and BZR1-mediated root cap maturation.

Since BR regulates transcriptional expression of diverse root-controlling genes in a BZR1 or BES1-dependent manner in this system, it was tested whether BZR1 or BES1 binds to the promoter of these genes *in vivo* by performing ChIP-qPCR analysis using *pBZR1::BZR1-YFP* and *p35S::BES1-GFP* plants. It was found that *BRAVO* and *ERF115* contain at least one or two BZR1 and/or BES1-binding motifs in their promoter regions (Fig. 6D), and this is consistent with the previous result showing that their expression was tightly controlled by BRI1/BZR1-mediated signalling (Fig. 1I). *PIN3*, *PIN4*, and *PIN7* genes contain at least one BZR1- and BES1-binding motif, and these results are strongly supported by a previous report demonstrating that BZR1 may bind to *PIN3*, *PIN4*, *PIN7* as evidenced by ChIP microarray (ChIP-chip) performed using transgenic *Arabidopsis* plants expressing the BZR1-CFP protein from the *BZR1* promoter (Sun *et al.*, 2010). The *BRN1* gene had at least one BZR1 and BES1-binding motif (Fig. 6D), but their effect on gene expression was different from each other (Fig. 6C). Taken together, these data indicate that BR-induced nuclear BZR1 regulates expression of several key root-controlling genes by binding to their promoters, which may be resulting in BR-induced modulation of root stem cell division and differentiation in the root tip area including columella cell layers.

## Discussion

A root is formed from a reservoir of undifferentiated cells, called root stem cells, in the root apical meristem. QC cells act as integrators for many processes and events that are requisites for root meristem establishment and maintenance. Previous studies proposed that QC cells might send short-range non-cell autonomous signals that help the initials remain in an undifferentiated state (van den Berg *et al.*, 1997; Scheres, 2007). CSC initials and the differentiated columella cells are longitudinally adjacent to the QC. Results presented here showed that BZR1- and BES1-mediated BR signalling pathways acted differentially in the regulation of the QC and CSC differentiation (see Supplementary Figure S3 at *JXB* online for a schematic model). Both pathways promoted ectopic QC cell division by down-regulating expression of *BRAVO* repressor while at the same time up-regulating *ERF115*, a positive regulator of QC division. In contrast, the effects of BRs in the regulation of maintenance and differentiation of the distal meristem were both BZR1-/BES1- and BL dose-dependent. The BRI1/BZR1-mediated BR signalling pathway inhibited the differentiation of distal CSCs, thus delaying the development of starch-filled columella cells. In contrast, BRI1/BES1-mediated BR signalling showed opposite effects on differentiation of the distal meristem, as previously reported (Gonzalez-Garcia *et al.*, 2011). Similarly, application of low concentrations of exogenous BL (~ ≤10^−10^ M) resulted in the inhibition of CSC differentiation, similar to the results of the BRI1/BZR1-mediated BR signalling pathway, while higher concentrations of BL (≥10^−9^ M) stimulated CSC differentiation as observed in BRI1/BES1-mediated BR signalling. Similarly, *bes1-D* seedlings were resistant to BRZ (a BR biosynthetic inhibitor) in both dark- and light-grown conditions (Yin *et al.*, 2002) whereas *bzr1-D* seedlings are resistant to BRZ in the dark but are hypersensitive in the light (Wang *et al.*, 2002). It has also been reported that BES1 acts as a transcriptional activator while BZR1 was shown to be a transcriptional repressor (He *et al.*, 2005; Yin *et al.*, 2005). It is conceivable that differences in BZR1-/BES1-dependent signalling dynamics provide the basis of the concentration-dependent differential responses. In fact, expression of distal CSC-regulating genes, such as *FEZ* and *BRN1*, seem to be differentially regulated in *bzr1-D* and *bes1-D* plants.

BRs interact with multiple hormone signalling pathways in the regulation of root growth and development (Lee *et al.*, 2013; Zhu *et al.*, 2013 for review). Regarding the interaction with auxin, BRs act synergistically with auxin to promote lateral root development by increasing acropetal auxin transport (Bao *et al.*, 2004), and enhance plant tropic responses by promoting the accumulation of PIN2 from the root tip to the elongation zone (Li *et al.*, 2005). Transcript profiling and ChIP-chip experiments have revealed that BZR1 binds to the promoters of many genes involved in auxin biosynthesis, transport, and signalling (Sun *et al.*, 2010). It has been demonstrated that BRs down-regulate transcriptional expression of *PINs* and change subcellular localization of their proteins, and thus may alter the auxin localization domain and subsequent root patterning. Supporting this model for BR-induced down-regulation of *PIN* genes in roots and the resulting antagonistic action of BR against auxin-induced CSC differentiation, it was recently proposed that oppositely patterned and antagonistic actions of BR and auxin maintain the stem cell balance and optimal root growth (Chaiwanon and Wang, 2015). Epidermis-driven BR signalling and activity is not associated with changes in the expression level of stele-localized *PINs* (*PIN1*, *PIN3*, and *PIN7*) (Hacham *et al.*, 2011), but modulates transcriptional expression of *PIN2* and *PIN4* and their accumulation in the epidermis/cortex and columella in a post-transcriptional manner (Hacham *et al.*, 2012). Thus, similar to the model proposed here, the authors proposed that BR-directed auxin distribution represents at least one mechanism that contributes to BR-mediated root growth. In particular, AtPIN4 mediates the sink-driven auxin gradient and the resulting auxin maximum in the QC and the columella initial, and thus controls signals for the QC to regulate auxin-driven root patterning. Consequently, the loss-of-function mutant *Atpin4* exhibits a divided QC, increased number of both CSC and CC layers, and increased root cap and swelling (Friml *et al.*, 2002).

Recent studies showed that auxin-induced repression of *WOX5* results in enhanced CSC differentiation (Ding and Friml, 2010). The BL-treated plants and *bzr1-D* plants with enhanced BR signalling used here exhibited a decreased level of auxin in the distal root area and increased expression of *WOX5*. Concomitantly, they exhibited reactivated QCs, an increased number of undifferentiated CSC layers, and a corresponding decreased number of columella cell layers. In summary, these findings indicate that the BZR1-mediated BR signalling pathway inhibits auxin-dependent distal stem cell differentiation in *Arabidopsis* roots through action that is antagonistic to the auxin/WOX5-dependent pathway. It is also possible that BR-driven nuclear BZR1 directly binds to the promoter of root-regulating genes such as *PINs*, *BRAVO*, *ERF115*, *FEZ*, and *BRN1*, and independently regulates QC division and the distal stem cell differentiation. Interestingly, abscisic acid (ABA) induces the expression of *WOX5* and thus maintains quiescence of the QC and suppresses differentiation of the columella initials (Zhang *et al.*, 2010). This phenomenon of ABA-induced *WOX5* expression and promotion of stem cell maintenance is opposite to the BZR1-mediated *WOX5* induction and reactivation of QC division in root tips that was observed in this study. Based on these apparently contradictory reports, it is highly plausible that differential regulation or mode of action of each hormone is involved in multiple cellular processes in distinctive root tissues or cell types, such as the QC, CSC, and CC. Therefore, it will be necessary to clarify their mode of action and interactomes by dissecting details of these regulatory frameworks and establishing their interaction with other hormones. It has been demonstrated that BZR1-mediated BR signalling and ETO1-promoted ethylene signalling pathways independently act on the QC reactivation event in a parallel manner. Supporting this observation, treatment of ethylene biosynthetic precursor ACC promoted QC division without promoting BZR1 nuclear accumulation in the QC (Chaiwanon and Wang, 2015).

Decoding the BR-dependent regulatory network in a cell-type specific manner remains a significant challenge, and any information will be useful in mapping BR interactomes with different pathways and uncovering how distinct tissues are coordinated (Fridman and Savaldi-Goldstein, 2013). Here, it has been shown that the effects of BRs in the regulation of QC and distal meristem activities are both BL dose- and BZR1-/BES1-dependent. Nonetheless, when and how BRs control stem cell maintenance and differentiation in roots remains largely unknown. Therefore, future studies must clearly dissect the connections among the dose-dependent perception of BR signals in a cell or tissue, the downstream signalling molecules, and the subsequent developmental patterning occurring in roots. Traditionally, an ARF-binding *DR5*-based auxin-inducible reporter (Ulmasov *et al.*, 1997; Barbez *et al.*, 2013) and an Aux/IAA-based auxin signalling sensor (DII-VENUS; Brunoud *et al.*, 2012) have been widely used to accurately map auxin response and distribution at high spatiotemporal resolution in a single-cell-based tobacco system and *in planta*. Thus, one useful approach would be to develop BR-specific sensors (reporters) and monitor real-time spatiotemporal changes in BR abundance in a cell or a tissue and perform computational modelling to establish BR distribution circuits during the developmental progress. In addition, the identification of BR-regulated target genes in a specific cell type or at a specific developmental time and clarifying their interactive regulation by other hormones will provide another level of understanding of BR-regulated meristem function in plants.

## Supplementary material

Supplementary data are available at *JXB* online.

Figure S1. Schematic diagram of the root SCN area.

Figure S2. Schematic diagram of potential BZR1- and BES1-binding sites (BRRE and E-box) and the PCR-amplified DNA fragments used for ChIP-qPCR analysis.

Figure S3. Schematic model explaining BR regulation of the QC maintenance and CSC differentiation.

Table S1. Primers used in quantitative real-time RT-PCR analysis.

Table S2. Primers used in ChIP-qPCR analysis.

Table S3. Division of QC in various BR mutants.

Supplementary Data
