# Supplementary material for: Brassinazole resistant 1 (BZR1)-dependent brassinosteroid signalling pathway leads to ectopic activation of quiescent cell division and suppresses columella stem cell differentiation
Source: J Exp Bot. 2015 Jul 1;66(15):4835–49. doi: 10.1093/jxb/erv316 (PMC4507784; doi:10.1093/jxb/erv316)
Supplement: Supplementary Data [file supp_66_15_4835__index.html]

Brassinazole resistant 1 (BZR1)-dependent brassinosteroid signalling pathway leads to ectopic activation of quiescent cell division and suppresses columella stem cell differentiation — Brassinazole resistant 1 (BZR1)-dependent brassinosteroid signalling pathway leads to ectopic activation of quiescent cell division and suppresses columella stem cell differentiation — Supplementary Data 

# Brassinazole resistant 1 (BZR1)-dependent brassinosteroid signalling pathway leads to ectopic activation of quiescent cell division and suppresses columella stem cell differentiation

## Supplementary Data

Data files

- Supplementary Data - Supplementary Data
