# Supplementary material for: Brassinazole resistant 1 (BZR1)-dependent brassinosteroid signalling pathway leads to ectopic activation of quiescent cell division and suppresses columella stem cell differentiation
Source: J Exp Bot. 2015 Jul 1;66(15):4835–49. doi: 10.1093/jxb/erv316 (PMC4507784; doi:10.1093/jxb/erv316)
Supplement: Supplementary Data [file supp_erv316_jexbot146894_file001.pdf]

BZR1-dependent brassinosteroid signaling pathway leads to ectopic activation of quiescent cell division and suppresses columella stem cell differentiation

Hak-Soo Lee, Yoon Kim, Giang Pham, Ju Won Kim, Ji-Hye Song, Yew Lee, Yong-Sic Hwang, Stanley J. Roux, Soo-Hwan Kim

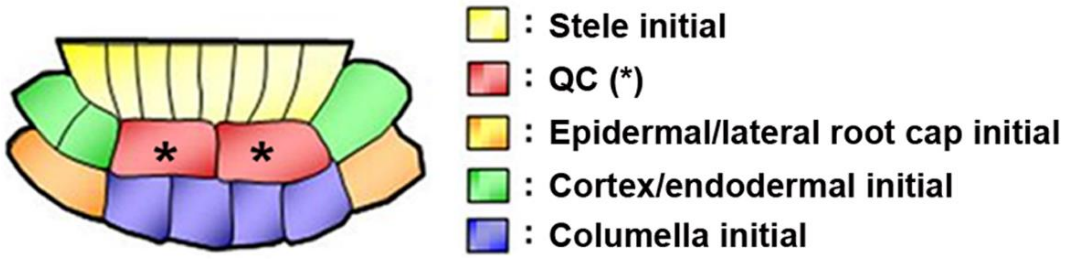

Supplementary Figure S1. Schematic diagram of the root SCN area.

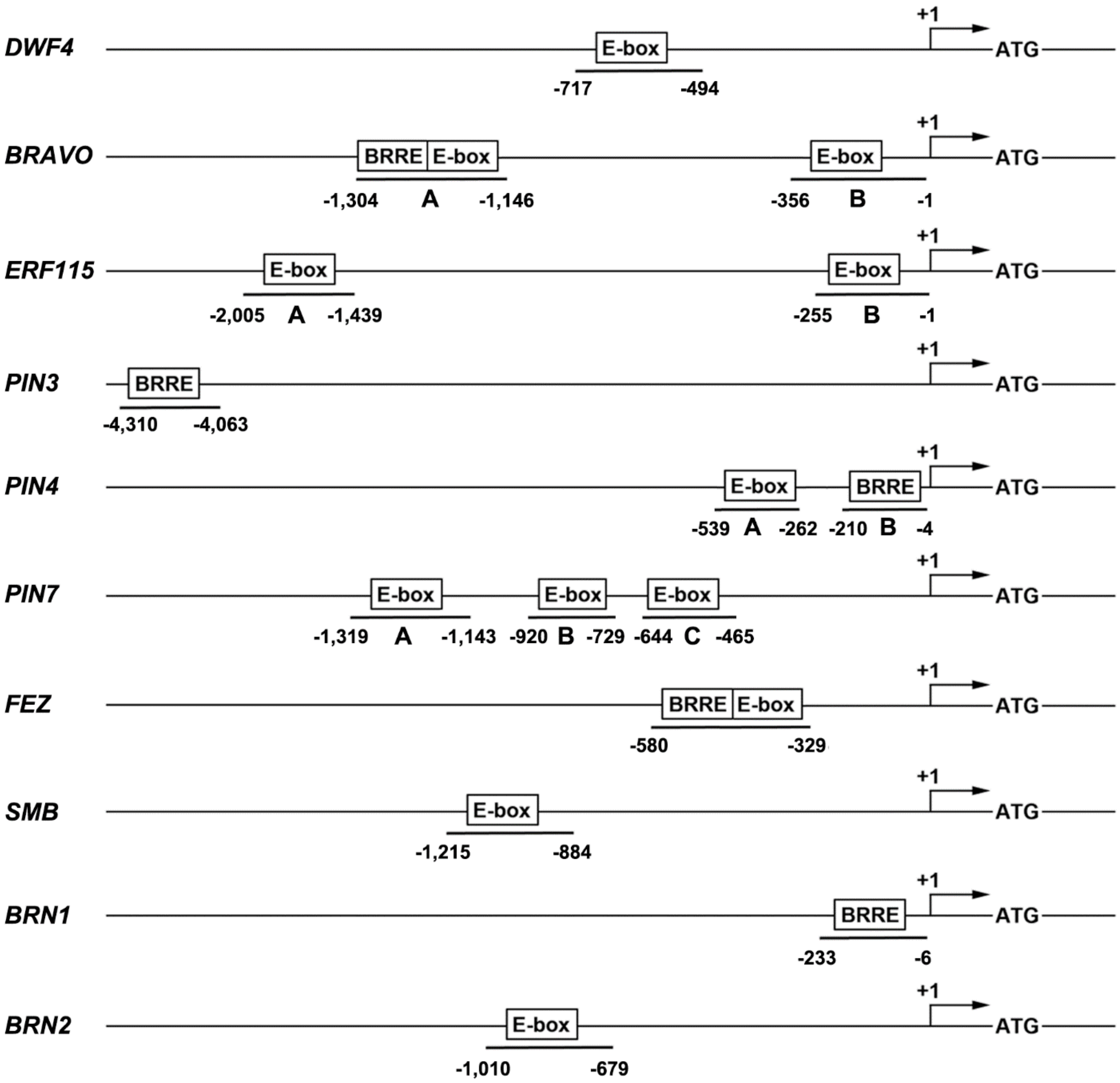

Supplemental Figure S2. Schematic diagram of potential BZR1- and BES1- binding sites (BRRE and E-box) and the PCR-amplified DNA fragments used for ChIP-qPCR analysis. 5' and 3' position of each fragment is indicated by numbers. +1 represents the transcriptional initiation site of the corresponding gene. Nucleotide positions and fragments lengths are not in scale.

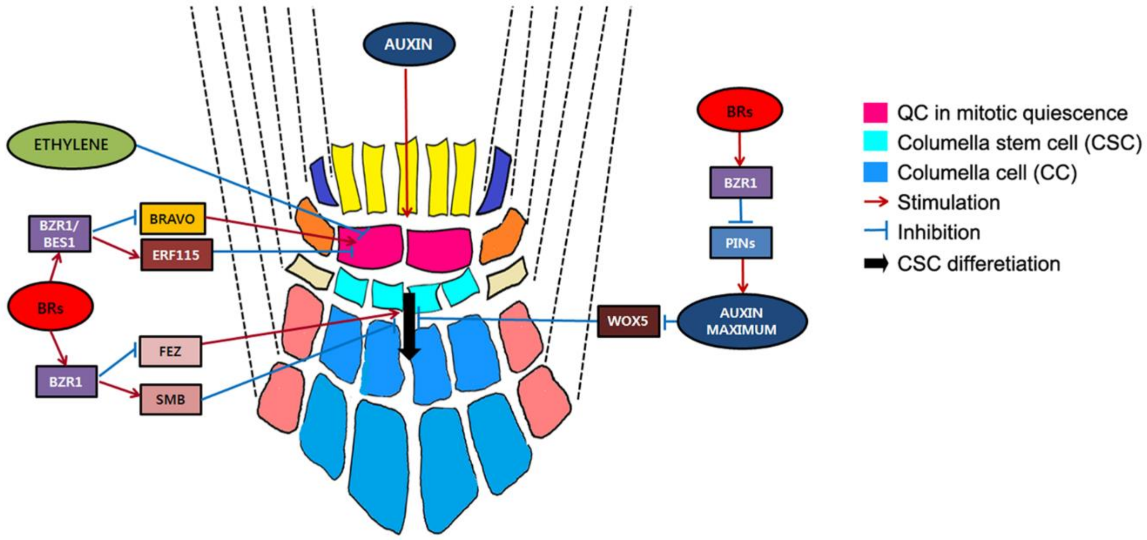

Supplemental Figure S3. A schematic model explaining brassinosteroid regulation of the QC maintenance and the columella stem cell (CSC) differentiation. Both BZR1-/BES1-mediated brassinosteroid signaling pathways promote mitotic reactivation of QC cells by inhibiting *BRAVO* (a negative regulator of QC division) and stimulating *ERF115* (a positive regulator of QC division) gene expression. In contrast, BRs regulate CSC differentiaion into columella cells (CCs) in a BZR1-/BES1-dependent manner. BZR1-mediated inhibitory BR action on PINs expression and activities provoke proximal movement of auxin maximum leading to WOX5 -mediated CSC differentiation into CCs. In other hand, BZR1 down-regulates gene expression of FEZ (encoding a protein promoting periclinal root cap-forming cell division), and this may result in another level of suppression for CSC differentiation into CCs. Ethylene pathway and BZR1-mediated BR signaling pathway acts independently on the QC reactivation.

**Supplemental Table S1. Primers used in quantitative real time RT-PCR analysis**		
Gene name	Locus	Primer set (5’ to 3’)
WOX5	At3g11260	TCCAACTCCAAGGTGGACAAAATGA
		ATGGCGGTGGATGTTCCATTTCAG
SCR	At3g54220	TTCTCACCCCCTCACTGAGTTTTTG
		GTTGTTGGTCGTGAGATTGCATGG
SHR	At4g37650	TGGGAAGAGAGTTTTCCAAGGACGA
		TCATCCGCCACCTCATCACTATACC
BRAVO	At5g17800	CGAGGACACTGGAGACCAACAGAAG
		CAG CGT TAT CGG TAC GAC CTG GA
ERF115	At5g07310	CAAATCCGCAGACTAATCCGCAAAC
		AGGAGGTGAAGAATCCCCAAAACG
PIN3	At1g70940	GGAGCACCTGACAACGATCAAGG
		CTCGGCGTCTTTTGGTCTCTCTG
PIN4	At2g01420	ATGTGCATCCCACGATTCTAAGCAC
		CAATCTCCGAGGCTCTCTCAAAAGC
PIN7	At1g23080	TTGGGCTCTTGTTGCTTTCAGGT
		CCGCTGGTCCAGTAAAGAATCTCAC
CUL3	At1g26830	TCCCGATCATCCGTCTTTCCTCT
		GCAGGACCATGTTGTACGCATTTC
ACS5	At5g65800	GCGATGCTTTCCTTTTGCCTACTC
		TTTCTGGGCTTGTTGGTAAGCTTGT
ETO1	At3g51770	TGCTGGATGCAGCTGTATGATCGTT
		GCTGCCTTTTGACAATTGAGCCGTA
FEZ	At1g26870	TCAGTTTGCAGCACCTTCATGTTTC
		CACCGTGGATGACGACCCTATCT
SMB	At1g26870	TCGGAAATGGGAGATAGAAACAACG
		TTGGAAGATCCCAGGGGTCATATTT
BRN1	At1g33280	CACGTGCAAGGCAGTAAGTGAATGG
		TCACCGCGCAATGAAAGCAGATTAG
BRN2	At4g10350	TCAAGCCAACCCTAGTGAAGATGGA
		TGGACTGTCTCGGTGCATAAAGCTA
CPD	At5g05690	GCGGTGTTTTCAGACGTGCAAT
		GAAAGTGCGAGCATCTTTGAAGTGG

**Supplemental Table S2. Primers used in ChIP-qPCR analysis**		
Gene name	Locus	Primer set (5' to 3')
UBC30	At5g56150	CAAATCCAAAACCCTAGAAACCGA
		AACGACGAAGATCAAGAACTGGGAA
PP2A	At1g69960	AGCAGCACAACCCTCAACAG
		CCAGATGTGCTAAAGACGGAG
DWF4	At3g50660	GTGTTTTCTGACTATTGAGGGG
		CGGTACGGTCTCAATCGGTTTA
BRAVO A	At5g17800	AAAATTTAAATTTAAACTAGTAGCAAAAAAT
		TTTTACTTATATACTATATTCAGTG
BRAVO B	At5g17800	AAAAAAAAAAAAAAATGATAAATAAAA
		GAGAGCACTTGAATGGCTTTTCACTG
ERF115 A	At5g07310	CGTTCTCGTCAACAAATCTGAAAATAC
		CAATCGAGAAACTGTTGTCTTTTTTTT
ERF115 B	At5g07310	TATGCAAAACTTCTGCTTGACGTTAA
		CTTTGCTAAAATCTTTAAACCTCTTT
PIN3	At1g70940	CTCCAATACTCGATCGTGAAGA
		GGATGATAGAGTGTGGATTGG
PIN4 A	At2g01420	CAAAAACAAAAAACAAAATAT
		AAAGTTGCAAAAGGAACTTTG
PIN4 B	At2g01420	GCACGACTATTCCATAAAACTGT
		GGATTCGGTGAAGAGGACTA
PIN7 A	At1g23080	CCAAACCATGAGCAGAATTGT
		CGTTTACACAATTATAATAGCAG
PIN7 B	At1g23080	CGGTCGCGGAAAGATCTTGA
		CCCAAGAAATCTCACTTTTAAG
PIN7 C	At1g23080	CGGCGAATATGATCTTGCATT
		GGGCCACTTAACGTATTACTAG
FEZ	At1g26870	CGATCGGCCCTTGTATCCTTTTAT
		AAAAATGCACTATCATCATATCGT
SMB	At1g26870	TAAGGAGCAAATATGGAACTTTA
		AGGATTAACTATACTAAGCAGTAA
BRN1	At1g33280	CTCCGGCGGCGTTGTCACCGGCCG
		GTTAATAAATGATCAATGTTTGTTTG
BRN2	At4g10350	TACATTAATATAACACATATTATT
		AGAAACACAAAAAGAAAAACCTTT

Supplemental Table S3. Division of QC in various brassinosteroid mutants

	Col	*bri1-116*	*bzr1-1D*	*bri1-116* /*bzr1-1D*	En-2	*bes1-D*
DAG 3	0/25 (0)[a]	0/32 (0)	0/24 (0)	0/28 (0)	0/25 (0)	0/31 (0)
DAG 7	2/38 (5)	0/26 (0)	14/28 (50)	10/27 (37)	0/23 (0)	21/25 (84)
DAG 14	5/28 (18)	2/22 (9)	24/32 (75)	20/29 (69)	2/21 (10)	33/33 (100)

[a]: Number of plants with periclinal division of QC / number of plants observed (percentage of plants with the reactivated QC). DAG: Day after germination.
